# Effect of obstructive sleep apnea–hypopnea syndrome on myocardial mechanics in obese patients

**DOI:** 10.3389/fcvm.2026.1672585

**Published:** 2026-04-01

**Authors:** Xiaoyan Kang, Jiping Xue, Min Zhao, Yanxia Zhang, Junwang Miao, Shuai Li, Chunsong Kang

**Affiliations:** Department of Ultrasonography, Third Hospital of Shanxi Medical University, Shanxi Bethune Hospital, Shanxi Academy of Medical Sciences, Tongji Shanxi Hospital, Taiyuan, Shanxi, China

**Keywords:** echocardiography, myocardial mechanics, obesity, obstructive sleep apnea–hypopnea syndrome, two-dimensional speckle tracking

## Abstract

**Objective:**

Obesity and obstructive sleep apnea-hypopnea syndrome (OSAHS) often coexist. Chronic intermittent hypoxia in OSAHS can compound cardiac remodeling, but the comprehensive impact of OSAHS severity on obese patients remains unclear.

**Methods:**

A total of 83 obese patients were included in this study and divided into the obesity-only group (27 cases) and the obesity-OSAHS group (56 cases) based on the presence of OSAHS. An additional 30 healthy individuals served as the control group. According to the apnea-hypopnea index (AHI), the obesity-OSAHS group was further classified into a mild group (30 cases), moderate group (16 cases), and severe group (10 cases). Echocardiographic and strain parameters were compared across these groups, and factors influencing global longitudinal strain (GLS) were analyzed.

**Results:**

Left ventricular GLS progressively declined across the control group (−21.28%), obesity-only group (−18.85%), and obesity-OSAHS group (−17.03%) (all P<0.05). Within the OSAHS group, GLS decreased with increasing severity: mild (−18.00%), moderate (−16.69%), and severe (−14.69%) (all *P* < 0.05). Structural cardiac remodeling was more pronounced in the severe group, with a left ventricular mass index (LVMI) value of 101.64 g/m2, compared with 87.11 g/m2 in the moderate group and 83.94 g/m2 in the mild group (*P* = 0.016). The AHI and LVMI were identified as independent predictors of reduced GLS.

**Conclusion:**

Obese patients exhibit impaired myocardial mechanics, which is further aggravated by OSAHS. With increasing OSAHS severity, GLS declines and myocardial dysfunction worsens. AHI and LVMI are independent risk factors for this dysfunction in obese patients with OSAHS.

## Introduction

]Obesity has emerged as a critical global public health challenge. Over the past four decades, its prevalence has more than doubled worldwide, currently affecting more than a billion individuals (1). It contributes to left ventricular remodeling and cardiac dysfunction through hemodynamic changes, neurohormonal abnormalities, and metabolic disturbances, establishing it as an independent risk factor for cardiovascular diseases (2, 3). Obstructive sleep apnea-hypopnea syndrome (OSAHS) is a common sleep disorder closely associated with obesity. It is defined by ≥30 episodes of apnea/hypopnea or an apnea–hypopnea index (AHI) of ≥5 events per hour during a 7-hour sleep period (4). Epidemiological studies indicate that a 10% weight gain is linked to a 32% increase in AHI, while a 10% weight loss can reduce AHI by 26% (5).

Obesity may cause OSAHS through various mechanisms, such as upper airway congestion caused by neck fat accumulation, and functional residual lung capacity reduction due to fat deposits around the chest cavity, abdomen and internal organs (6). OSAHS can lead to chronic intermittent hypoxia, autonomic dysfunction, endothelial damage, and oxidative stress—all of which contribute to adverse cardiovascular events (7). Early detection of myocardial mechanical abnormalities in patients with OSAHS is helpful for identifying subclinical left ventricular systolic dysfunction (8). Global longitudinal strain (GLS), measured by two-dimensional speckle tracking echocardiography, serves as a sensitive early indicator of left ventricular systolic function, enabling quantitative assessment of myocardial mechanics and prediction of cardiovascular events (9). As obesity rates continue to rise, the incidence of OSAHS is also steadily increasing. Although studies have confirmed that both obesity and OSAHS can impair myocardial mechanics, the impact of their combination on myocardial function in obese patients remains unclear. This article explores the effects of OSAHS and its varying severity on myocardial mechanics in obese individuals using two-dimensional speckle tracking imaging technology. The goal is to provide a basis for preventing and treating myocardial damage in obese patients with OSAHS and to inform proactive clinical intervention and treatment strategies.

## Materials and methods

### Study participants

This retrospective analysis included 92 obese patients who were admitted to Shanxi Bethune Hospital from September 2021 to October 2023 and were scheduled to undergo bariatric surgery. After excluding 9 patients because of inadequate image quality, 83 patients were enrolled: 21 male and 62 female patients aged 16–51 years (mean, 30.08 ± 7.88 years). The patients were divided into an obesity-only group (*n* = 27) and an obesity–OSAHS group (*n* = 56). The obesity–OSAHS group was further stratified by AHI: mild (5 ≤ AHI < 15, *n* = 30), moderate (15 ≤ AHI < 30, *n* = 16), and severe (AHI ≥ 30, *n* = 10). The individual characteristics of the severe OSAHS group are shown in Supplementary Table S1. Apnea was defined as airflow reductions ≥90% from baseline lasting ≥10 s. Hypopnea was defined as a ≥30% reduction in airflow lasting ≥10 s and associated with ≥4% oxyhemoglobin desaturation (10). The control group comprised 30 age- and sex-matched healthy individuals without snoring or a history of cardiovascular disease. This study was conducted following the Helsinki Declaration and approved by and approved by the Ethics Committee of Shanxi Bethune Hospital Affiliated to Shanxi Medical University (YXLL-2022-034), and all participants signed an informed consent form.

The inclusion criteria were as follows: obesity defined according to Chinese population standards as a body mass index (BMI) of ≥28 kg/m^2^ (11), diagnosis of OSAHS with an AHI of ≥5 events/h (7), and age between 18 and 65 years. The exclusion criteria were diabetes, hypertension, or hyperlipidemia; dilated or hypertrophic cardiomyopathy, rheumatic heart disease, congenital heart disease, myocardial infarction, arrhythmia, unstable angina pectoris, or a history of percutaneous coronary intervention or coronary artery bypass grafting; chronic obstructive pulmonary disease, interstitial lung disease, asthma, or other chronic respiratory diseases; and poor image quality that did not meet analysis requirements.

### Clinical data collection

Demographic parameters (age, sex, height, weight) and cardiovascular-metabolic indices—including heart rate, systolic blood pressure, diastolic blood pressure, fasting plasma glucose, total cholesterol, high-density lipoprotein cholesterol, triglycerides, and low-density lipoprotein cholesterol—were recorded. BMI and body surface area were calculated using the following standard formulas:

$$\mathrm{BMI}=\mathrm{weight}\;(\mathrm{kg})/\mathrm{height}\;(m{)}^{2}$$

Bodysurfacearea=0.0061×height+0.0128×weight−0.1529

### Polysomnography

All participants underwent overnight polysomnography monitoring (21:00–07:00) using an Alice 5 system (Respironics Inc., Murrysville, PA, USA) in the hospital's bariatric surgery department, having avoided strong tea, coffee, alcohol, and sedatives beforehand. The AHI was calculated as the average number of apnea or hypopnea events per hour of sleep.

### Ultrasound equipment and examination methods

A GE Vivid E9 color Doppler ultrasound system (GE HealthCare, Chicago, IL, USA) with an M5S transducer (1.5–4.5 MHz) and EchoPAC analysis software version 203 (GE HealthCare) was used.

Each patient underwent a standard echocardiographic examination (12). Prior to image acquisition, participants were instructed to rest for 5 min and were connected to an electrocardiogram in the left lateral position. M-mode echocardiography was performed from the long-axis view of the left ventricle to measure interventricular septal thickness at end-diastole (IVSTd), left ventricular posterior wall thickness at end-diastole (LVPWTd), left ventricular end-diastolic diameter (LVEDD), and left ventricular end-systolic diameter (LVESD). Simpson's biplane method was used to manually outline the left ventricular endocardial boundary, and the left ventricular ejection fraction was automatically calculated by the instrument. Early diastolic mitral peak flow velocity (E) was measured using pulsed Doppler in the apical four-chamber view, and early diastolic mitral annular average velocity (e′) in the septal and lateral walls was obtained via tissue Doppler imaging. The average E/e′ ratio was then calculated. Dynamic images were acquired with the highest possible frame rate (frame rate 60–80 frames/s) and optimal image quality and stored in three standard transthoracic apical views (apical four-, two-, and three-chamber views) for offline analysis. All measurements were taken over at least three cardiac cycles, and average values were used. Left ventricular mass (LVM) was calculated using the following formula:

$$\;\mathrm{LVMI}(g)=0.8\times 1.04\times [{\left(\mathrm{IVSD}+\mathrm{LVPWD}+\mathrm{LVEDD}\right)}^{3}-\mathrm{LVED}D^{3}]+0.6.$$

The LVM index (LVMI) was obtained by adjusting LVM for body surface area: LVMI=LVM/Bodysurfacearea.

### Image analysis

Image analysis was performed on the EchoPAC workstation by importing each patient's two-dimensional gray-scale dynamic images. Two experienced physicians, blinded to the clinical data, carried out the analysis. The Q-Analysis strain analysis program was accessed, and the “2D Strain” function was selected once the left ventricular endocardial surface was clearly visible in the apical four-, two-, and three-chamber views. Manual adjustments to the tracing line were made as needed to ensure accurate tracking of all left ventricular segments. After selecting “Approve,” the software automatically generated the GLS value of the left ventricular myocardium along with a 17-segment bull's-eye map of longitudinal strain.

### Repeatability test

Echocardiographic images from 20 randomly selected individuals with obesity were analyzed to assess measurement repeatability. Two experienced physicians independently performed the GLS analysis using a double-blind approach, without knowledge of the participants' group assignments, to minimize bias. Inter-observer repeatability was also evaluated. After a 1-week interval, one of the observers reanalyzed the same images to assess intraobserver repeatability.

### Statistical analysis

SPSS 26.0 statistical software (IBM Corp., Armonk, NY, USA) was used for data analysis. The Kolmogorov–Smirnov test was applied to assess the normality of measurement data. Normally distributed data are presented as mean ± standard deviation. One-way analysis of variance was used to compare parameters between groups, with the least significant difference t test employed for further pairwise comparisons. For non-normally distributed data, values are expressed as the median and interquartile range. The Kruskal–Wallis H test was used for group comparisons, with multiple comparison corrections performed using the Bonferroni method. Categorical data are presented as frequency (number of cases), and comparisons between groups were made using the *χ*^2^ test. Univariate regression analysis was used to examine the relationship between GLS and both clinical and echocardiographic parameters. Variables with *P* < 0.10 in the univariate analysis were then included in a multivariate linear regression model to identify factors influencing GLS. Repeatability was assessed using Bland–Altman plots and intraclass correlation coefficients (ICCs). Statistical significance was set at *P* < 0.05.

## Results

### Comparison of general clinical parameters among groups

The BMI, systolic blood pressure, diastolic blood pressure, and fasting plasma glucose of the obesity-only group and the obesity–OSAHS group were all higher than those of the control group, while high-density lipoprotein cholesterol was lower than in the control group. The AHI of the obesity–OSAHS group was higher than that of both the obesity-only and control groups, and the heart rate was also higher than in the control group. The differences were all statistically significant (all *P* < 0.05). These results reflect that compared with the control group, obese patients exhibit more pronounced hemodynamic changes and metabolic abnormalities.

In the obesity–OSAHS group, the AHI values gradually increased in the mild, moderate, and severe subgroups, and the differences were statistically significant (all *P* < 0.05) (Table 1).

**Table 1:** Comparison of general clinical parameters among groups.

Variables		Control group (*n* = 30)	Obe-only group (*n* = 27)	Obe-OSA group (*n* = 56)	*χ*^2^*/F/*H value	*P* value	Effect size	Mil group (*n* = 30)	Obe-OSA group	Sev group (*n* = 10)	*χ*^2^*/F/*H value	*P* value	Effect size
									Mod group (*n* = 16)				
Age, years		32.47 ± 3.35	29.41 ± 8.16	30.41 ± 7.80	2.587	0.084	0.026	28.63 ± 7.57	31.75 ± 8.71	33.60 ± 5.97	1.912	0.158	0.067
Male, *n* (%)		12 (40%)	4 (14.81%)	17 (30.36%)	4.431	0.109	0.198	5 (16.67%)	8 (50.00%)	4 (40.00%)	6.059	0.053	
Smoke	1	5	7	8	2.748	0.601	0.156	2	3	3	4.675	0.322	0.289
	2	8	4	11				5	4	2			
	3	17	16	37				23	9	5			
Duration of obesity, years		–	6.0 (3.0, 10.0)	8.0 (4.0, 10.8)	0.751	0.626	0.009	5.50 (2.0, 10.0)	7.00 (4.3, 10.0)	10.500 (6.3, 16.3)	2.252	0.324	0.040
BMI, kg/m^2^		21.67 ± 0.81	39.80 ± 5.55^a^	42.07 ± 6.32^a^	409.350	<0.001	0.740	41.08 ± 5.92	42.47 ± 6.28	44.38 ± 7.49	1.067	0.351	0.039
AHI, bpm		2.05 (1.13, 3.33)	2.50 (1.80, 3.70)	13.95^ab^ (10.75, 22.73)	84.258	<0.001	0.746	11.34 ± 1.88	20.37 ± 3.80^c^	65.66 ± 26.58^cd^	57.626	<0.001	0.770
SBP, mmHg		116.10 ± 2.99	122.15 ± 5.92^a^	125.20 ± 8.16^a^	33.287	<0.001	0.251	124.13 ± 8.61	126.06 ± 8.37	127.00 ± 6.51	0.580	0.563	0.021
DBP, mmHg		73.77 ± 3.38	77.81 ± 5.70^a^	78.54 ± 6.86^a^	11.418	<0.001	0.109	77.17 ± 6.60	78.94 ± 7.63	82.00 ± 5.56	1.964	0.150	0.069
HR, bpm		80.13 ± 3.49	81.67 ± 5.76	83.18 ± 7.41^a^	3.444	0.038	0.042	82.47 ± 8.41	83.25 ± 5.47	85.20 ± 7.15	0.502	0.608	0.019
FPG, mmol/L		4.33 ± 0.37	5.10 ± 0.38^a^	5.25 ± 0.47^a^	47.603	<0.001	0.464	5.13 ± 0.44	5.37 ± 0.48	5.39 ± 0.47	1.983	0.148	0.070
TG, mmol/L		1.04 ± 0.23	1.06 ± 0.27	1.16 ± 0.25	2.758	0.068	0.048	1.14 ± 0.22	1.18 ± 0.28	1.19 ± 0.29	0.219	0.804	0.008
TC, mmol/L		4.08 ± 0.55	3.99 ± 0.48	4.11 ± 0.61	0.371	0.691	0.007	4.06 ± 0.57	4.08 ± 0.69	4.30 ± 0.65	0.616	0.544	0.023
HDL-C, mmol/L		1.35 ± 0.10	1.09 ± 0.17^a^	1.05 ± 0.17^a^	60.725	<0.001	0.408	1.09 ± 0.20	1.01 ± 0.13	1.00 ± 0.14	1.572	0.217	0.056
LDL-C, mmol/L		2.62 ± 0.27	2.75 ± 0.36	2.72 ± 0.36	1.111	0.333	0.020	2.73 ± 0.35	2.71 ± 0.43	2.68 ± 0.31	0.072	0.931	0.003

Obe-only, obesity-only; Obe-OSA, obesity-OSAHS; Mil, mild; Mod, moderate; Sev, severe; Smoke: Code 1 indicates regular smoking (smoking more than 1 cigarette per day, continuously or cumulatively for 6 months or longer); Code 2 indicates occasional smoking; Code 3 indicates never smoking; BMI, body mass index; AHI, apnea–hypopnea index; SBP, systolic blood pressure; DBP, diastolic blood pressure; HR, heart rate; FPG, fasting plasma glucose; TG, triglycerides; TC, total cholesterol; HDL-C, high-density lipoprotein cholesterol; LDL-C, low-density lipoprotein cholesterol.

^a^ *P* < 0.05 compared with control group.

^b^ *P* < 0.05 compared with the obesity-only group.

^c^ *P* < 0.05 compared with mild group.

^d^ *P* < 0.05 compared with moderate group. Effect size: partial eta squared (*p η*^2^) or Phi coefficient (for *p η*^2^: 0.01 = small, 0.06 = moderate, 0.14 = large; for Phi: 0.1 = small, 0.3 = moderate, 0.5 = large); 1 mmHg = 0.133 kPa.

### Comparison of echocardiography parameters and GLS among groups

Compared with the control group, both the obesity-only and obesity–OSAHS groups showed higher IVSTd, LVPWTd, LVEDD, LVESD, LVMI, and average E/e′ values (all *P* < 0.05). In addition, IVSTd, LVPWTd, and LVMI were higher in the obesity–OSAHS group than in the obesity-only group (all *P* < 0.05).

Within the obesity–OSAHS group, the IVSTd, LVPWTd, and LVMI in the severe subgroup were greater than those in the mild and moderate subgroups (all *P* < 0.05). The mean E/e′ in the severe group was higher than in the mild group (*P* < 0.05).

**Figure 1:**
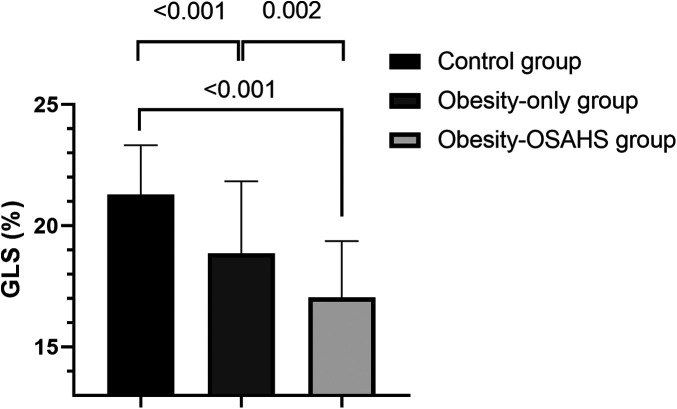
Comparison of GLS among groups. GLS in the control group, obesity-only group, and obesity–OSAHS group decreased in sequence.

GLS values decreased sequentially across the control group, the obesity-only group, and the obesity–OSAHS group (*P* < 0.05), indicating that myocardial mechanics were impaired in obese patients and further deteriorated in those with OSAHS (Table 2 and Figure 1).

**Table 2:** Comparison of echocardiography parameters and GLS among groups.

Cardiac parameters	Control group (*n* = 30)	Obe-only group (*n* = 27)	Obe-OSA group (*n* = 56)	*χ*^2^*/F* value	*P* value	Effect size	Obe-OSA group					
							Mil group (*n* = 30)	Mod group (*n* = 16)	Sev group (*n* = 10)	*χ*^2^*/F* value	*P* value	Effect size
IVSTd, mm	8.67 ± 1.32	9.56 ± 1.15^a^	10.41 ± 1.52^ab^	15.674	<0.001	0.222	10.00 ± 1.41	10.38 ± 1.45	11.70 ± 1.34^cd^	5.434	0.007	0.170
LVPWTd,mm	8.27 ± 1.34	9.15 ± 1.29^a^	10.16 ± 1.50^ab^	18.253	<0.001	0.249	9.63 ± 1.27	10.31 ± 1.49	11.50 ± 1.35^cd^	7.288	0.002	0.216
LVEDD, mm	42.23 ± 3.13	49.81 ± 3.56^a^	50.29 ± 2.20^a^	79.012	<0.001	0.611	50.17 ± 2.00	50.38 ± 2.31	50.50 ± 2.76	0.101	0.904	0.004
LVESD, mm	27.67 ± 2.66	32.33 ± 3.83^a^	33.07 ± 2.62^a^	41.294	<0.001	0.383	32.93 ± 2.74	33.19 ± 2.34	33.30 ± 2.91	0.092	0.912	0.003
LVEF (%)	65.07 ± 3.35	65.04 ± 5.43	64.64 ± 4.31	0.140	0.869	0.002	65.23 ± 4.52	63.63 ± 4.16	64.50 ± 3.98	0.725	0.489	0.027
Average E/e′	6.61 ± 1.37	8.20 ± 1.25^a^	8.86 ± 1.65^a^	22.237	<0.001	0.288	8.36 ± 1.70	8.98 ± 1.26	10.17 ± 1.40^c^	5.234	0.008	0.165
LVMI, g/m^2^	70.14 ± 15.26	80.12 ± 17.48^a^	88.00 ± 17.26^ab^	11.136	<0.001	0.168	83.94 ± 16.28	87.11 ± 12.94	101.64 ± 20.59^cd^	4.476	0.016	0.145
GLS (%)	−21.28 ± 2.04	−18.85 ± 2.99^a^	−17.03 ± 2.33^ab^	30.043	<0.001	0.353	−18.00 ± 1.90	−16.69 ± 2.35^c^	−14.69 ± 1.67^cd^	10.568	<0.001	0.285

IVSTd, interventricular septal thickness at end-diastole; LVPWTd, left ventricular posterior wall thickness at end-diastole; LVEDD, left ventricular end-diastolic diameter; LVESD, left ventricular end-systolic diameter; LVEF, left ventricular ejection fraction; Average E/e′, ratio of peak early diastolic mitral flow velocity (E) to average mitral annular velocity at lateral wall and septum (e′); LVMI, left ventricular mass index; GLS, global longitudinal strain.

^a^ *P* < 0.05 compared with control group.

^b^ *P* < 0.05 compared with obesity-only group.

^c^ *P* < 0.05 compared with mild group.

^d^ *P* < 0.05 compared with moderate group. Effect size: partial eta squared (*p η*^2^) (for *p η*^2^: 0.01 = small, 0.06 = moderate, 0.14 = large).

**Figure 2:**
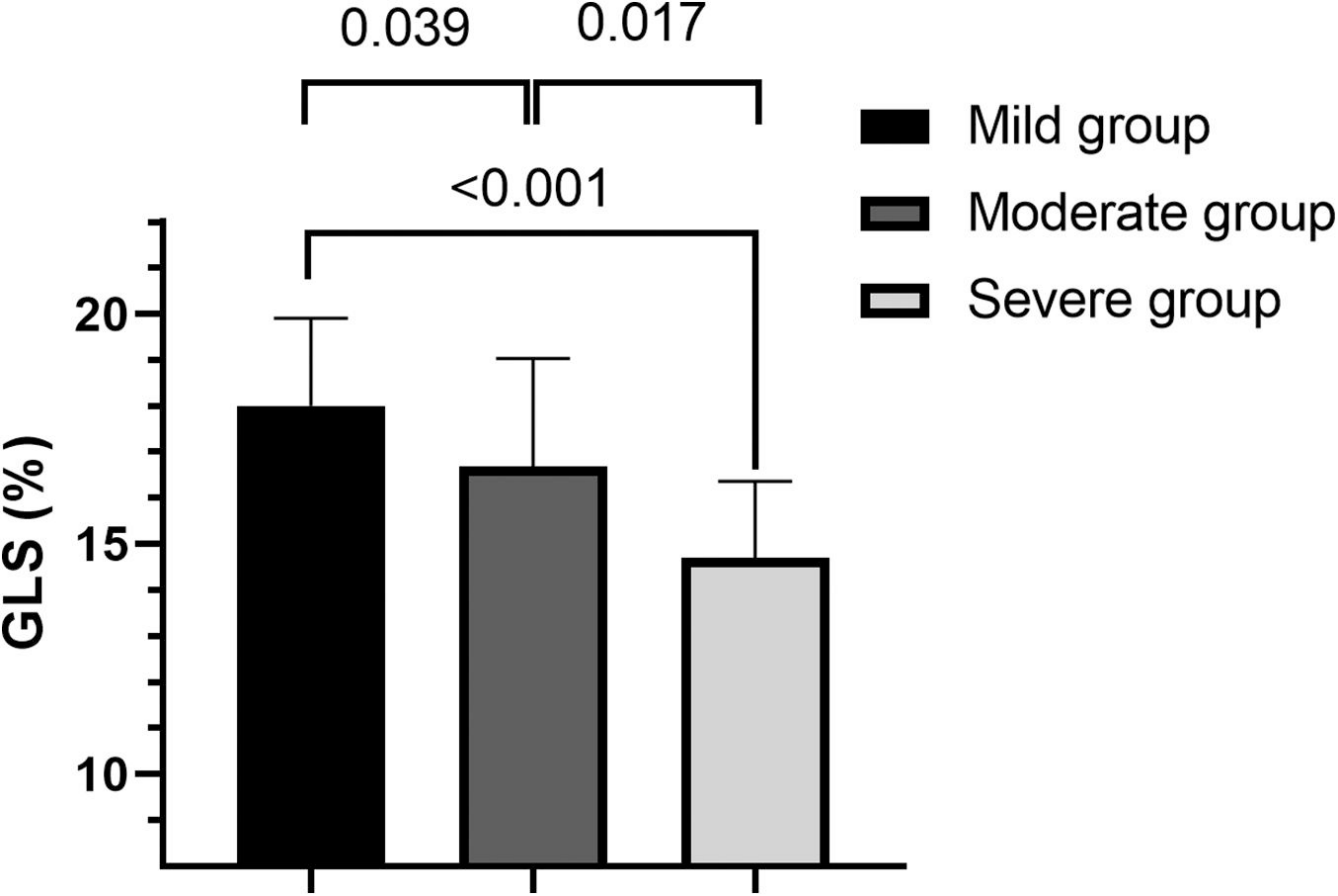
Comparison of GLS among obese patients with varying OSAHS severity. Among obese patients, GLS progressively decreased with increasing severity of OSAHS.

Within the obesity–OSAHS group, GLS decreased significantly with increasing OSAHS severity (mild > moderate > severe), with all differences statistically significant (all *P* < 0.05), suggesting that left ventricular myocardial mechanics progressively worsened as OSAHS severity increased (Figure 2). The GLS bull's-eye diagram of the three groups demonstrated GLS decreased sequentially (Supplementary Figure S1).

### Univariate regression analysis of factors influencing GLS

All 83 obese patients included in the study were analyzed as a single cohort. Univariate regression analysis was performed to assess the relationship between GLS and both clinical and echocardiographic parameters. GLS was significantly associated with sex, AHI, duration of obesity, LVMI, average E/e′, and HDL-C (*P* < 0.10) (Table 3).

**Table 3:** Univariate regression analysis of factors influencing GLS.

Variables	GLS				
	B	SE	*Β*	*t* value	*P* value
Age	−0.029	0.038	−0.086	−0.781	0.437
Gender	1.640	0.656	0.268	2.499	0.014
Smoke	0.495	0.375	0.145	1.319	0.191
Duration of obesity	−0.133	0.045	−0.313	−2.962	0.001
BMI	0.005	0.049	0.012	0.105	0.916
AHI	−0.061	0.012	−0.486	−4.999	<0.001
SBP	−0.050	0.039	−0.141	−1.280	0.204
HR	−0.056	0.043	−0.145	−1.315	0.192
FPG	0.191	0.673	0.032	0.284	0.777
TC	0.151	0.519	0.032	0.291	0.772
HDL-C	3.593	1.686	0.230	2.132	0.036
LDL-C	−0.218	0.823	−0.029	−0.265	0.792
LVPWD	−0.624	0.185	−0.350	−3.361	0.001
IVSD	−0.687	0.189	−0.375	−3.639	0.001
LVEDD	−0.153	0.109	−0.154	−1.403	0.164
LVESD	−0.094	0.097	−0.108	−0.974	0.333
LVEF	0.023	0.064	0.041	0.366	0.715
Average E/e′	−0.373	0.187	−0.216	−1.995	0.049
LVMI	−0.056	0.016	−0.368	−3.562	0.001

### Multivariate regression analysis of factors influencing GLS

Using GLS as the dependent variable, variables with *P* < 0.10 in the univariate regression analysis were entered into a multivariate linear regression model to identify factors influencing GLS. The results showed that AHI and LVMI were independent risk factors for reduced GLS in obese patients (Table 4).

**Table 4:** Multivariate regression analysis of factors influencing GLS.

Variables	B	SE	*β*	*t* value	*P* value	95% CI of B	VIF
Constant	19.085	2.585		7.383	<0.001	13.937 to 24.234	
AHI	−0.038	0.014	−0.305	−2.834	0.006	−0.065 to −0.011	1.363
Gender	0.919	0.602	0.150	1.527	0.131	−0.280 to 2.119	1.136
Duration of obesity	−0.073	0.041	−0.172	−1.770	0.081	−0.156 to 0.009	1.118
HDL-C	1.445	1.525	0.093	0.948	0.346	−1.592 to 4.483	1.127
Average E/e′	−0.032	0.172	−0.019	−0.187	0.852	−0.376 to 0.311	1.180
LVMI	−0.036	0.015	−0.237	−2.372	0.020	−0.066 to −0.006	1.175

### Repeatability study

The mean difference in GLS measurements between the two physicians for the same participants was 0.07 ± 0.48, and the ICC value was 0.986. The mean difference for repeated GLS measurements by the same physician after a 1-week interval was 0.04 ± 0.39, and the ICC value was 0.991. Bland–Altman analysis and ICCs showed that the differences in repeated GLS measurements were consistent with their respective means, indicating good interobserver and intraobserver agreement.

## Discussion

This study evaluated changes in cardiac structure and function in obese patients with OSAHS. The findings indicate that obese patients exhibit myocardial mechanical impairment, which is further exacerbated by the presence of OSAHS. Furthermore, the degree of impairment progressively worsens as the severity of OSAHS increases. The AHI and LVMI are independent risk factors for reduced GLS in obese patients.

Our study showed that that the obesity-only group had significantly higher IVSTd, LVPWTd, LVEDD, LVESD, LVMI, and mean E/e′ along with lower GLS compared with the control group, indicating obesity-related impairments in left ventricular structure and myocardial mechanics. Previous studies have shown that even after excluding other factors such as hypertension, diabetes, and dyslipidemia, there remains a significant association between obesity and cardiovascular disease risk (13). The long-term increase in volume load in obese patients contributes to left ventricular enlargement. Additionally, activation of the renin–angiotensin–aldosterone system triggered by sympathetic nervous system stimulation—even in obese individuals with normal blood pressure—can increase left ventricular afterload (14), leading to left ventricular systolic dysfunction and GLS reduction ultimately (15).

There were no significant differences in cardiovascular risk factors such as BMI, blood pressure, blood sugar, and blood lipids between obesity-only group and obesity–OSAHS group. However, the IVSTd, LVPWTd, and LVMI of OSAHS group were higher than obesity-only patients, and the GLS was lower than the latter. This suggests that when OSAHS and obesity coexist, myocardial structural and mechanical damage is further aggravated, exhibiting a superimposed effect of the two conditions on early left ventricular myocardial dysfunction. Studies have shown that both OSAHS and obesity can cause myocardial remodeling and early impairment of cardiac function through sympathetic nervous system activation, insulin resistance, systemic inflammation, and oxidative stress (16). Research by D'Andrea et al. (17) showed that obese patients with OSAHS had significantly increased IVSTd, LVPWTd, and LVMI, along with a reduced GLS (−23.4% ± 4.3% vs. −15.8% ± 2.6%), compared with the control group. These findings are consistent with our research results. However, it should be noted that some OSAHS patients in their study had comorbidities such as hypertension, hyperglycemia, and hyperlipidemia, a situation prevalent in most studies, which makes it challenging to confirm the independent impact of OSAHS itself on cardiac structure and function (18). Therefore, our study cohort was restricted to obese patients without other comorbidities, thereby avoiding the confounding effects of other factors on cardiac function.

In addition, we further investigated the cardiac structure and function of obese patients with varying severities of OSAHS. The results showed that compared with the mild group, the moderate group had a significant decrease in GLS, while no significant differences were observed in other parameters apart from AHI. However, the echocardiographic parameters IVSTd, LVPWTd, and LVMI were higher in the severe group than in the mild and moderate groups, and GLS decreased further. In a study of 244 overweight patients with OSAHS, Ma et al. (19) classified participants into a mild group (AHI < 15) and a moderate-to-severe group (AHI ≥ 15). They found a significant reduction in GLS in the moderate-to-severe group compared with that in the mild group (−18.2% ± 2.1% vs. −20.1% ± 2.4%), indicating that the severity of OSAHS can exacerbate GLS damage independently of overweight. In addition, Wang et al. (20) documented a progressive decline in GLS across mild, moderate, and severe groups (−17.8% ± 1.5%, −15.9% ± 1.4%, and −14.8% ± 1.5%). Varghese et al. (21) also reported that patients with severe OSAHS exhibited significantly impaired GLS compared to the control group (−15% ± 1.8% vs. −19% ± 1.6%). The gradual deterioration of GLS with increasing disease severity was further demonstrated in the meta-analysis by Tadic et al. (22). However, due to differences in population characteristics among the above studies, there are certain variations in the GLS measurements, but the trends are generally consistent. Previous studies have indicated that GLS is influenced by numerous factors, such as age, sex, blood pressure, BMI, and comorbidities, with the reference range of −24% to −16% (23, 24). Nevertheless, this serves only as a general guide. In practice, conclusive findings depend on comparisons with a well-matched control group and the careful exclusion of confounding variables.

The multiple linear regression analysis showed that both AHI and LVMI were significantly associated with reduced left ventricular GLS. In early OSAHS, a hypothesized mechanism driven by rising AHI involves intermittent hypoxia, which subjects cardiomyocytes to repeated hypoxia-reperfusion cycles. This impairs mitochondrial function, leading to an inadequate energy supply, progressing to cardiomyocyte injury and diminished mechanical performance (16). Compared with the mild group, the moderate group in this study exhibited an increased AHI and decreased GLS. Therefore, the decrease in GLS in the moderate group may be more influenced by AHI, which may be related to the study population. Our cohort comprised individuals scheduled for bariatric surgery—a demographic typically presenting with younger age, a relatively higher proportion of women, and a shorter disease duration. Echocardiographic assessment revealed no significant myocardial remodeling in the moderate group. Given this finding, the observed reduction in GLS is more likely a direct consequence of the elevated AHI rather than an association with increased LVMI. As OSAHS worsens, we propose that the ensuing myocardial mechanical dysfunction may be mediated not only by direct hypoxic damage but also by synergistic effects of sympathetic overactivity and elevated vascular resistance from severe nocturnal intrathoracic pressure fluctuations and chronic intermittent hypoxia (15, 25, 26). The impact of LVMI on GLS has been well established: increased LVMI is associated with cardiomyocyte hypertrophy, myocardial interstitial fibrosis, and subsequent systolic dysfunction (27, 28).

This study has several limitations. First, although the sample size met basic statistical requirements, it was relatively small overall, particularly in the severe OSAHS group, which included only 10 cases. This small sample may not reflect the broader severe OSAHS population and limits the precision of parameter estimates, reducing the reliability of the conclusions. Second, the sample was predominantly female, resulting in an unbalanced sex distribution. In addition, the strict exclusion criteria may introduce selection bias and further limit the generalizability of the findings. Moreover, this study was designed as a single-center cross-sectional investigation and failed to incorporate several critical variables, including the duration of OSAHS, treatment status, physical activity levels, neck circumference and sleep parameters. In the future, studies should expand the sample size (especially increasing representation of severe cases and male patients), adopt multicenter longitudinal designs with detailed pre- and post-treatment data, and refine inclusion and exclusion criteria to strengthen the study design and verify these conclusions.

## Conclusion

Myocardial mechanical function in obese patients is impaired, and this impairment is further exacerbated when obesity is combined with OSAHS. As OSAHS severity increases, GLS progressively decreases, indicating worsening myocardial mechanical damage. AHI and LVMI were identified as independent risk factors for impaired myocardial mechanical function in obese patients with OSAHS. Notably, in moderate OSAHS, AHI may be the main contributor to myocardial dysfunction. These findings suggest that clinicians should prioritize individualized assessment and stratified management in obese patients with moderate to severe OSAHS.

## Data Availability

The original contributions presented in the study are included in the article/Supplementary Material, further inquiries can be directed to the corresponding author/s.
