# Supplementary material for: Effect of obstructive sleep apnea–hypopnea syndrome on myocardial mechanics in obese patients
Source: Front Cardiovasc Med. 2026 Apr 1;13:1672585. doi: 10.3389/fcvm.2026.1672585 (PMC13078984; doi:10.3389/fcvm.2026.1672585)
Supplement: Supplementary file 1 [file Supplementaryfile1.docx]

Supplementary Material

## Supplementary Figure


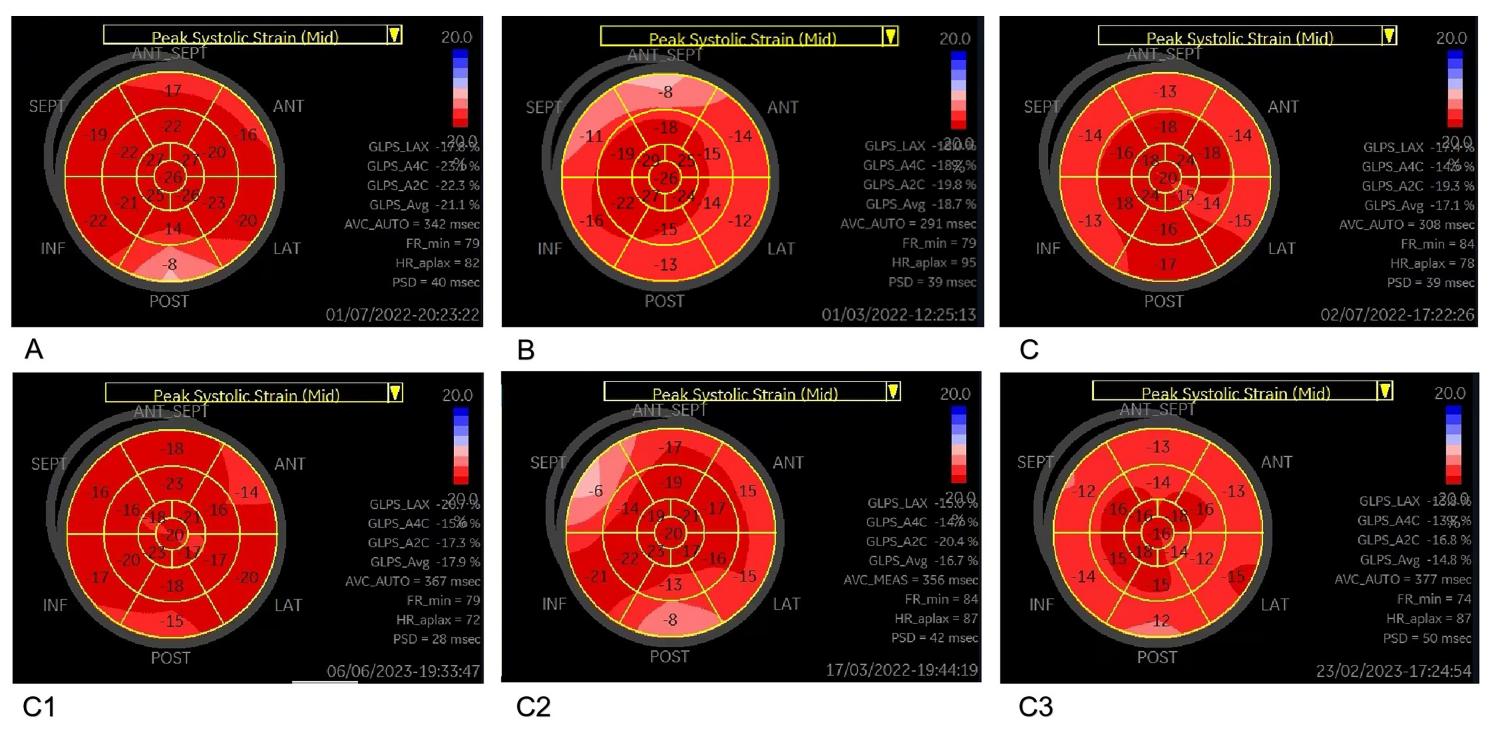


**Supplementary Figure 1.** GLS bull’s-eye diagram. GLS decreased sequentially in the control group, obesity-only group, and obesity–OSAHS group, and within the OSAHS group, GLS declined further with increasing severity. A: Control group, GLS=-21.1%; B: Obesity-only group, GLS=-18.7%; C: Obesity–OSAHS group, GLS=-17.1%; C1: Mild group, GLS=-17.9%; C2: Moderate group, GLS=-16.7%; C3: Severe group, GLS=-14.8%
