# Supplementary material for: Effect of obstructive sleep apnea–hypopnea syndrome on myocardial mechanics in obese patients
Source: Front Cardiovasc Med. 2026 Apr 1;13:1672585. doi: 10.3389/fcvm.2026.1672585 (PMC13078984; doi:10.3389/fcvm.2026.1672585)
Supplement: Supplementary file 2 [file Table1.docx]

Supplementary table 1. Individual characteristics of the severe OSAHS group

|  | AHI  (bpm) | Gender  （female=1,man=2） | Age  (years) | BMI  (kg/m2) | SBP  (mmHg) | DBP  (mmHg) | HR  (bpm) | FPG  (mmol/L) | TG  (mmol/L) | TC  (mmol/L) | HDL-C  (mmol/L) | LDL-C  (mmol/L) | Duration of obesity  (years) | Smoke |
| --- | --- | --- | --- | --- | --- | --- | --- | --- | --- | --- | --- | --- | --- | --- |
| 1 | 31.6 | 1 | 30 | 45.47 | 133 | 88 | 84 | 4.86 | 0.81 | 4.25 | 0.94 | 2.92 | 8 | 2 |
| 2 | 90.7 | 1 | 38 | 36.85 | 125 | 84 | 93 | 4.79 | 1.44 | 4.46 | 1.2 | 2.84 | 15 | 3 |
| 3 | 45.6 | 1 | 40 | 46.95 | 135 | 89 | 76 | 5.8 | 1.39 | 4.46 | 1 | 3.04 | 1 | 3 |
| 4 | 112 | 2 | 42 | 42.40 | 134 | 86 | 87 | 6.07 | 0.92 | 3.68 | 0.83 | 2.67 | 20 | 1 |
| 5 | 63.8 | 1 | 23 | 58.20 | 122 | 81 | 92 | 4.92 | 1.3 | 3.38 | 1.01 | 2.18 | 1 | 3 |
| 6 | 33.8 | 2 | 38 | 33.46 | 128 | 81 | 82 | 5.31 | 1.58 | 5.07 | 0.95 | 3.15 | 10 | 2 |
| 7 | 60.4 | 2 | 28 | 49.03 | 130 | 85 | 78 | 5.6 | 0.68 | 3.86 | 1.24 | 2.4 | 10 | 1 |
| 8 | 92.3 | 1 | 34 | 35.57 | 122 | 77 | 82 | 5.63 | 1.25 | 5.13 | 1.06 | 2.58 | 22 | 3 |
| 9 | 51.9 | 1 | 33 | 46.89 | 114 | 71 | 80 | 5.9 | 1.21 | 3.64 | 0.94 | 2.39 | 11 | 3 |
| 10 | 74.5 | 2 | 30 | 48.95 | 127 | 78 | 98 | 4.99 | 1.29 | 5.08 | 0.84 | 2.66 | 14 | 1 |

Smoke: Code 1 indicates regular smoking (smoking more than 1 cigarette per day, continuously or cumulatively for 6 months or longer); Code 2 indicates occasional smoking; Code 3 indicates never smoking
